# The cyclic AMP phosphodiesterase RegA critically regulates encystation in social and pathogenic amoebas^☆^

**DOI:** 10.1016/j.cellsig.2013.10.008

**Published:** 2014-02

**Authors:** Qingyou Du, Christina Schilde, Elin Birgersson, Zhi-hui Chen, Stuart McElroy, Pauline Schaap

**Affiliations:** College of Life Sciences, University of Dundee, Dundee DD15EH, UK

**Keywords:** PDE, phosphodiesterase, cAMP, 3′5′-adenosine monophosphate, PKA, cAMP dependent protein kinase, ACG, adenylate cyclase G, AcrA, adenylate cyclase R, MRSA, methicillin resistant *Streptococcus aureus*, *Acas*, *Acanthamoeba castellani*, *Ddis*, *Dictyostelium discoideum*, *Ppal*, *Polysphondylium pallidum*, KO, knock-out, RI, random integrant, Stress signalling, Encystation, cAMP-phosphodiesterase, Sensor histidine kinase, *Acanthamoeba* keratitis

## Abstract

Amoebas survive environmental stress by differentiating into encapsulated cysts. As cysts, pathogenic amoebas resist antibiotics, which particularly counteracts treatment of vision-destroying *Acanthamoeba* keratitis. Limited genetic tractability of amoeba pathogens has left their encystation mechanisms unexplored. The social amoeba *Dictyostelium discoideum* forms spores in multicellular fruiting bodies to survive starvation, while other dictyostelids, such as *Polysphondylium pallidum* can additionally encyst as single cells. Sporulation is induced by cAMP acting on PKA, with the cAMP phosphodiesterase RegA critically regulating cAMP levels. We show here that RegA is deeply conserved in social and pathogenic amoebas and that deletion of the *RegA* gene in *P. pallidum* causes precocious encystation and prevents cyst germination. We heterologously expressed and characterized *Acanthamoeba RegA* and performed a compound screen to identify RegA inhibitors. Two effective inhibitors increased cAMP levels and triggered *Acanthamoeba* encystation. Our results show that RegA critically regulates Amoebozoan encystation and that components of the cAMP signalling pathway could be effective targets for therapeutic intervention with encystation.

## Highlights

• Amoebas differentiate into dormant encapsulated cysts when exposed to environmental stress
• Encystation renders pathogenic amoebas resistant to antibiotics and biocides
• The social amoeba *Polysphondylium pallidum* is amenable to genetic approaches to resolve encystation mechanisms
• The cAMP phosphodiesterase RegA and the sensor histidine kinases that regulate RegA activity are deeply conserved
• RegA regulates encystation in *P.pallidum* and in the pathogen *Acanthamoeba castellani*

## 1 Introduction

Differentiation into dormant encapsulated cysts, or encystation, is the main differentiation process of amoebas and most other unicellular eukaryotes. Encystation is triggered by starvation and other environmental challenges [1], and as cysts the organisms can withstand these challenges for months up to years [2]. Encystation is of considerable medical importance, because cysts of pathogenic amoebas are impervious to immune attack and treatment with antibiotics or antiseptics [3], [4], [5], [6], [7]. This is a particular problem in the treatment of eye infections caused by opportunistic pathogens, such as *Acanthamoeba castellani.* This common inhabitant of soil and surface waters also colonizes other habitats, such as drinking water and air-conditioning ducts [8]. The eye infections are most prevalent in careless contact lens wearers, with outbreaks being caused by substandard lens cleaning fluids [9], [10]. The infections require months of painful treatment with a cocktail of antibiotics and antiseptics. They are often recurrent because the therapeutic challenge causes the amoebas to encyst, and frequently leads to the loss of the cornea or eye [7], [11], [12]. Amoebozoan cysts are also exploited by bacterial pathogens, such as *Legionella*, MRSA and *Vibrio cholerae,* as vectors for long time survival and air-borne dispersal [13], [14], [15]. Lack of gene disruption procedures applicable to free-living Amoebozoa, has left the mechanisms that control encystation largely unexplored.

The social amoeba *Dictyostelium discoideum* (*Ddis*), also a member of Amoebozoa, is a popular genetic model system for investigating problems in cell- and developmental biology. It has adopted a novel survival strategy in response to nutrient stress: the starving amoebas aggregate to form multicellular fruiting bodies, in which a proportion of cells differentiates into dormant walled spores, while the remainder differentiate into a stalk that supports the spore mass. Spore differentiation is triggered by extracellular cAMP acting on G-protein coupled receptors [16], [17] and intracellular cAMP acting on cAMP-dependent protein kinase (PKA) [18], [19]. *Ddis* does not form cysts, but in other Dictyostelia, such as *Polysphondylium pallidum* (*Ppal*), amoebas still encyst individually under wet and dark conditions that are unfavorable for aggregation and fruiting body formation. Spores have a much thicker wall than cysts and are more dehydrated [20], which probably makes them even more environmentally resilient than cysts.

The adenylate cyclases ACG and AcrA have overlapping roles in synthesizing cAMP for activation of PKA in sporulation [21], [22]. Particularly ACG then also acts in the spore to prevent germination under conditions that do not favor the proliferation of amoebas [23]. However, it is actually the cAMP phosphodiesterase RegA that plays the most pivotal role in controlling intracellular cAMP levels. The phosphodiesterase (PDE) activity of RegA is controlled by phosphorylation of its intrinsic response regulator domain by sensor histidine kinases/phosphatases. There are 15 sensor histidine kinases/phosphatases in the *Ddis* genome and at least four of these are receptors for signals that control the timely formation and germination of spores in an intricate network of communication between the maturing spore and stalk cells [24], [25], [26], [27], [28], [29], [30], [31], [32].

In this work we used the genetically tractable encysting Dictyostelid *Ppal* to investigate whether RegA critically regulates encystation. We show that this is the case and then identified and expressed a *RegA* gene from *Acanthamoeba castellani (Acas).* By using a pharmacological approach, we also established an essential role for RegA in encystation of this pathogen.

## 2 Materials and methods

### 2.1 Gene disruption, cloning and expression

#### 2.1.1 *P.pallidum RegA* gene disruption

To disrupt *P.pallidum* (*Ppal*) *RegA1*, two *RegA1* fragments comprising base pairs 139–1333 (A) and 1896–2833 (B), respectively, were amplified from *Ppal* PN500 genomic DNA, using primer pairs PpRegAI5′/PpRegAI3′ and PpRegAII5′/PpRegAII3′ (Table S1). The primers generated KpnI/BamHI and HindIII/HindIII restriction sites, flanking the two fragments. After HindIII digestion, fragment B was inserted into HindIII site vector pLox-NeoI, which, after selection of a construct with the appropiate orientation of fragment B, was further complemented after KpnI/BamHI digestion with KpnI/BamHI digested fragment A, yielding pRegA1KO (Supplementary Fig. S2A).

*Ppal* PN500 cells were transformed by electroporation with the linearized vector pRegA1KO according to established procedures [33]. Genomic DNA was isolated from G418 resistant clones and screened by two PCR reactions and Southern blot to diagnose *RegA1* gene disruption by homologous recombination (Fig. S2B,C). Four knock-out (KO) clones and four random integrants (RIs) were identified from two independent transformations.

#### 2.1.2 Cloning and expression of Acas RegA

The partially assembled *Acas* genome http://blast.hgsc.bcm.tmc.edu/blast.hgsc?organism=AcastellaniNeff was queried by tBlastn with *Ddis* RegA, yielding hits on 3 contigs, which after assembly yielded about 3.3 kb of coding sequence homologous to the query sequence, but containing many introns. To identify intron positions, we amplified a cDNA from *Acas* mRNA by reverse transcripion PCR. Total *Acas* RNA was isolated using the Qiagen RNeasy Mini Kit and reverse transcribed with SuperScript III First-Strand Synthesis System (Invitrogen, Paisley, UK), using primers AcRegAF and AcRegAR, that contained NheI and EcoRI sites respectively, followed by cDNA amplification with Phusion High-Fidelity DNA Polymerase (NEB, Ipswich, MA). The cDNA was cloned after NheI/EcoRI digestion into similarly digested pET28a (Novagen, Leuven, Belgium), yielding plasmid pET-AcRegA, in which *Acas* RegA is fused at the N-terminus to a hexahis-tag. The DNA sequence was determined from three clones and showed an open reading frame of 1863 bp.

To obtain *Acas* RegA protein, plasmid pET-AcRegA was transformed into *E.coli* BL21DE3. Bacteria were grown overnight at 37 °C in LB containing 30 μg/ml kanamycin. The culture was then diluted 1:40 in LB, incubated for 2 h at 30 °C and supplemented with 1 mM IPTG. After 4 h, cells were lysed using BugBuster® Protein Extraction Reagent (Novagen), the *Acas* RegA his-tag fusion protein was purified using Ni-NTA His.Bind® Resin (Novagen) and stored at − 80 °C.

### 2.2 Cell growth, development and encystation

#### 2.2.1 Growth and development

*Ppal,* strain PN500, was routinely grown in association with *Klebsiella aerogenes* on 1/5th SM agar and when appropriate in HL5 axenic medium (Table S3). Strain PN500 is naturally axenic and was further trained to grow effectively in HL5 by alternating growth from spores on HL5, and fruiting body formation on non-nutrient (NN) agar (Table S3) for a few months. For multicellular development, *Ppal* cells were harvested in 10 mM Na/K-phosphate, pH 6.5 (PB), washed free from bacteria and incubated at 10^6^ cells/cm^2^ and 22 °C on NN agar. *Acas,* strain Neff, was grown in AC medium (Table S3) at 21 °C.

#### 2.2.2 Encystation

For quantification of growth and encystation, *Ppal* cells were inoculated in HL5 at 3 × 10^5^ cells/ml and shaken at 150 rpm and 21 °C. Aliquots of 1 ml were sampled at regular intervals, centrifuged at 1000 × *g* for 1 min, and resuspended in 50 μl PB containing 0.001% calcofluor (which reacts to cellulose in the cyst wall). Total amoeba and cyst densities were determined by counting cells in a haemocytometer under phase contrast and UV illumination, respectively. 100–500 cells were counted for each time point.

*Acas* encystation was induced by incubating amoebas at 5 × 10^5^ cells/ml in starvation buffer (SB) (Table S3) [34]. Amoeba and cyst cell densities were determined at regular intervals, as described above.

### 2.3 Enzyme and cAMP assays

#### 2.3.1 Phosphodiesterase

To measure cAMP PDE activity, 0.02 μg of purified RegA was incubated for 30 min at 22 °C with 10 nM [2,8-^3^H]-cAMP (Perkin Elmer, Waltham, MA) and 1 mM MgCl_2_ in PB, with unlabelled cAMP, cGMP or PDE inhibitors as indicated. Reactions were terminated by boiling and [2,8-^3^H] 5′AMP was hydrolysed further with the 5′nucleotidase contained in 10 μg of *Naja messambica* snake venom (SA venom suppliers, Louis Trichardt, South Africa) to [2,8-^3^H]adenosine, which was separated from [2,8-^3^H]-cAMP by adsorption of the latter to Dowex anion exchange resin [35], and measured by scintillation counting.

#### 2.3.2 Cellular cAMP

To measure cellular cAMP levels, pellets of 10^7^ *Acas* cells were resuspended in 50 μl PB and lysed with 50 μl 3.5% perchloric acid. Samples were neutralized by adding 25 μl 50% saturated KHCO_3_ and 40 μl cAMP assay buffer (4 mM EDTA in 150 mM K-phosphate, pH 7.5) and centrifuged for 5 min at 13,200 × *g*. cAMP was assayed in 40 μl supernatant. The pellet was resuspended in 500 μl 0.1 M NaOH and assayed for protein.

## 3 Results

### 3.1 Deep conservation of RegA

**Fig. 1:**
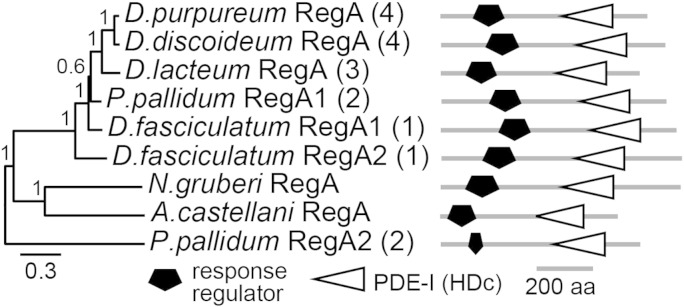
**RegA identification**. RegA homologs were retrieved by BLASTp search of all eukaryote sequences in Genbank and by tBLASTn search of ongoing *D.lacteum* (http://sacgb.fli-leibniz.de) and *A.castellanii (Acas)* (http://www.hgsc.bcm.tmc.edu) genome sequencing projects using *Ddis* RegA as bait. The *Acas* RegA protein sequence was deduced from reverse transcribed mRNA. Protein sequences were aligned using M-coffee [58] and regions that were not unambiguously aligned were deleted. Phylogenetic relationships between aligned sequences were determined by Bayesian inference [59] run for 1 million generations, using a mixed amino acid model, with rate variation between sites estimated by a gamma distribution with a proportion of invariable sites. The posterior probabilities of tree nodes are indicated and the tree is annotated with the functional domain architecture of the proteins. Numbers between brackets denote from which of the four major taxon groups the Dictyostelid sequences were derived [42]. Black and grey scale bars represent number of substitutions per site and protein length in amino-acids (aa), respectively.

All Genbank eukaryotic genes and amoebozoan genomes that were close to completion were screened for the presence of *Ddis* RegA homologs, which contained both the response regulator domain [36] and the HDc-type phosphodiesterase (PDE) domain [37] that characterize RegA [24], [25], [38]. One or two copies of RegA were detected in the genomes of *Dictyostelium purpureum* [39], *Dictyostelium lacteum* (Schaap, P. and Gloeckner, G. unpublished), *Ppal* and *Dictyostelium fasciculatum* [40], which, with *Ddis* [41], represent the four major groups of Dictyostelia [42]. A single *RegA* gene was found in the *Acas* genome [43] and in the genome of *Naegleria gruberi* [44]. The *RegA* homologs have a similar domain architecture across species (Fig. 1) with the response regulator domain followed by the PDE domain. The second *Ppal RegA* gene (*RegA2*) is highly derived (Fig. 1) and lacks several essential residues in both the response regulator and catalytic domain (Supplementary Fig. S1).

### 3.2 Disruption of the *P.pallidum* RegA gene

#### 3.2.1 Developmental phenotype

**Fig. 2:**
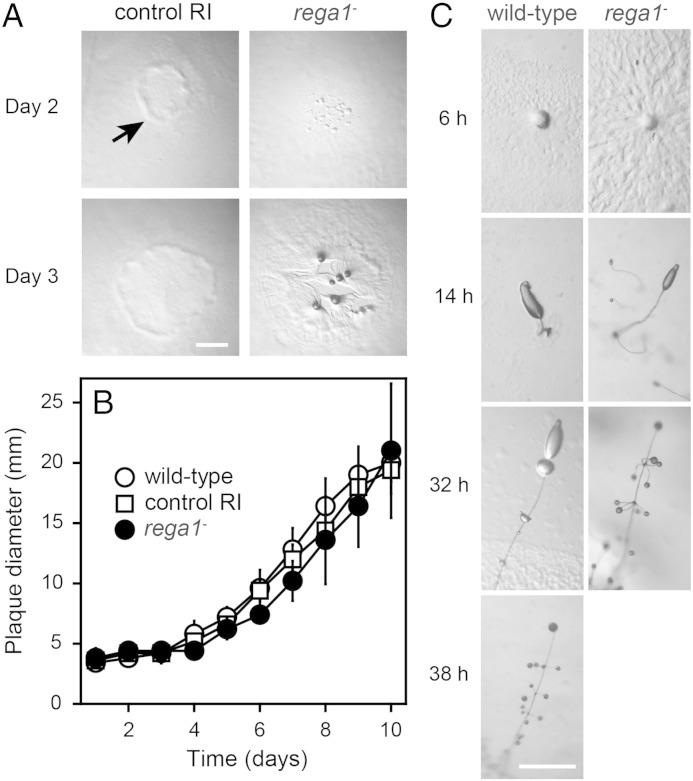
***P. pallidum regA1* knock-out phenotype.** *A/B. Growth on bacteria.* 10^5^ cells of the *Ppal rega1-*strain, KO38, the random integrant strain RI15 and wild-type cells were inoculated as 1 μl droplets on *K.aerogenes* lawns in quadruplicate and incubated at 22 °C under ambient light. KO and RI plaques were photographed at 2 and 3 days of incubation (A) and the diameter of all plaques was measured daily with a ruler for 10 days (B). Means and SD of measurements are presented. The arrow indicates the growth front. Bar: 1 mm. *C. Fruiting body morphogenesis. rega1-*KO38 and wild-type cells were harvested from *K.aerogenes* lawns before bacteria were cleared, plated on non-nutrient agar and incubated at 22 °C under ambient light. Developing structures were photographed at 2 h intervals. Bar: 0.5 mm.

To assess a possible role of RegA in encystation and a conserved role in sporulation, we abrogated the *Ppal RegA1* gene by homologous recombination (Fig. S2). *rega1-*knockout (KO) and control random integrant (RI) clones were obtained from two independent transformations and all KO clones showed the same phenotype, while the RI clones were mostly identical to wild-type cells. When plated as colonies on bacterial lawns, the *rega1-*cells formed rather diffuse plaques without a clear feeding front (Fig. 2A). A feeding front is formed by wild-type cells and the control RI strain (arrow), because the amoebas from cleared areas are attracted to bacteria at the periphery of the plaque. Instead, the *rega1-*cells initiated aggregation much earlier than control cells, with aggregates then attracting amoebas from regions where bacteria were not yet cleared. The KO and RI plaques increased in diameter at the same rate (Fig. 1B), suggesting that the proliferation of *rega1-*amoebas on bacteria is otherwise normal.

When pre-grown cells were developed on non-nutrient agar, the aggregation of *rega1-*cells was not obviously accelerated, but subsequent development into fruiting bodies proceeded at least seven hours faster than in wild-type cells. The morphology of the fruiting structures was otherwise normal (Fig. 2C). The *Ddis rega-*mutant also displays accelerated development, but it matures its spores before the stalk is properly formed, which results in spores sitting at the stalk base [24].

#### 3.2.2 Encystation

**Fig. 3:**
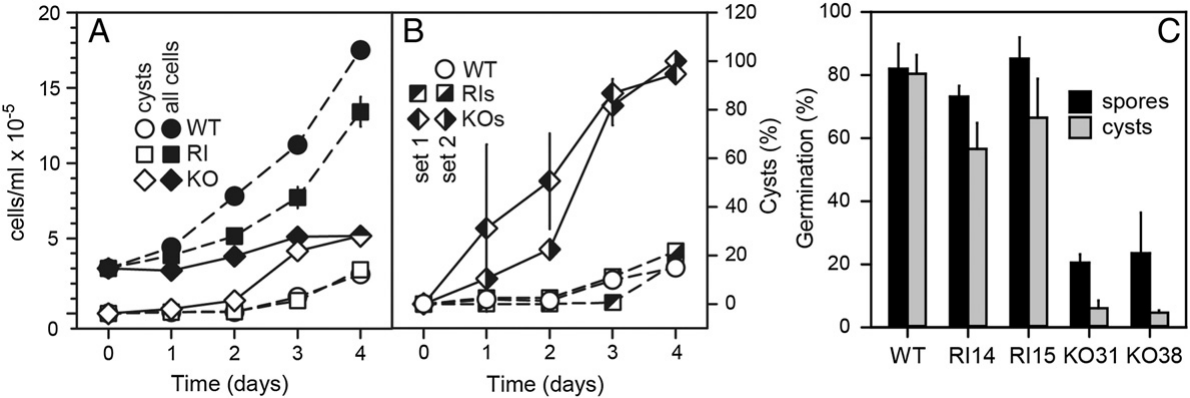
**Proliferation, encystation and germination in *rega1-*mutants**. A*. Proliferation*. *Ppal* wild type (WT), KO and random integrant (RI) strains were inoculated in HL5 liquid culture medium at 3 × 10^5^ cells/ml. Aliquots were stained with Calcofluor at daily intervals to stain cyst walls, and cyst and total cell numbers were counted over a 4 day period. Wild-type cells and means and SD of two RI (RI14, RI15) and two KO (KO31, KO38) strains are presented. B. *Encystation*. The experiment was repeated with two RI (RI11,RI12) and two KO (KO8,KO10) from another transformation (set 1, see Fig. S2) and the data from both sets were recalculated into percentage of encysted cells. Means and SD of two strains from each set are presented. C. *Cyst and spore germination*. *Ppal* wild-type, KO and RI spores were harvested from 5-day old fruiting bodies. For cysts, wild-type, KO and RI cells were grown to stationary phase in HL5. Cultures were supplemented with 0.25 M sorbitol and incubated for 3 more days to allow mature cysts to form. Cysts (grey bars) or spores (black bars) were treated for 10 min with 0.1% Triton-X100 to lyse amoeboid cells and washed with PB. Cysts and spores were plated with *K. aerogenes* over 10 plates each, with 50 spores/cysts per plate, and emerging *Ppal* colonies were counted after 3–4 days. Means and SD of 2 experiments are presented.

When *Ppal* cells starve under submerged conditions, they encyst individually instead of aggregating. This also occurs when *Ppal* cells are cultured on liquid axenic media at the time that the culture reaches stationary phase. We noticed that the *rega1-*strains proliferated poorly in axenic medium, and investigated amoeba growth and encystation systematically in all KO and RI strains. The control RI strains seemed to proliferate somewhat slower than wild-type *Ppal*, but the difference was not statistically significant. For both wild-type and RI strains only a small percentage of cells encysted after 3 days, when the culture approached stationary phase. The KO strains proliferated significantly slower than both wild-type *Ppal* and the RI strains (*P* = 0.002, Mann–Whitney rank sum test [45]), but already started to encyst after 1 day of culture, with cells almost fully encysted at 3 days (Fig. 3B). Because the *rega1-*cells proliferated normally on solid substratum (Fig. 2A), their proliferation defect in liquid culture is most likely due to precocious encystation.

#### 3.2.3 Cyst and spore germination

In *Ddis,* intracellular cAMP acting on PKA is essential to maintain dormancy [23], [46] and loss of RegA prevents spore germination [24]. To investigate whether this is also the case in *Ppal* and whether RegA1 additionally controls cyst germination, we investigated spore and cyst germination in the *Ppal rega1-*KO and RI cells. Fig. 3C shows that 75–85% of wild-type and RI spores, and 55–80% of cysts germinated within a three day period, but this was reduced to 20% for spores and 5% for cysts for the *rega1-*KO mutants. The reduction in both spore and cyst germination efficiency in the *rega1-*KO strains was statistically significant (*P* = 0.01, Mann–Whitney rank sum test). Combined, the data show that *Ppal RegA1* normally acts to prevent both precocious encystation and precocious multicellular development and to enable spore and cyst germination.

### 3.3 Cloning and characterization of *Acanthamoeba castellani RegA*

No gene knock-out strategies have been established for the opportunistic pathogen *Acanthamoeba castellani* and we therefore sought to functionally analyze *Acas* RegA and to identify effective inhibitors by a heterologous expression approach. The *Acas RegA* coding sequence was initially assembled from sequences, homologous to *Ddis RegA*, that were distributed over three contigs. To assign a gene model and to prepare an *Acas RegA* expression construct, we reverse-transcribed total *Acas* RNA and sequenced the *RegA* cDNA. This showed that the 3293 bp *Acas RegA* gene contained 12 introns and encoded a 621 aa protein.

**Fig. 4:**
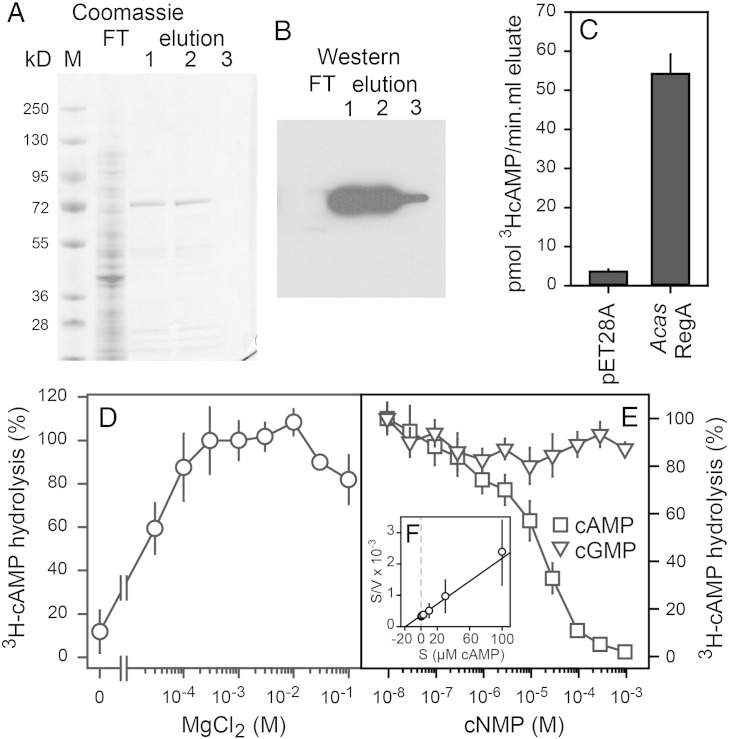
**Heterologous expression and characterization of *Acas* RegA**. A/B. *Heterologous expression.* The *Acas* RegA cDNA was fused to a hexa-his tag in vector pET28a, expressed in *E.coli,* and purified by Ni^+^ chromatography. The column flow-through (FT) and three fractions eluted with 250 mM imidazole were size-fractionated by SDS-PAGE (A). Western blots of the size-fractionated proteins (B) were incubated with 1:2000 diluted mouse anti his-tag antibody and 1:5000 diluted peroxidase conjugated goat-anti-mouse IgG, followed by peroxidase detection. C. *Acas RegA activity*. 1 μl aliquots of the combined 250 mM imidazole eluate fractions of expressed *Acas* RegA and combined eluates obtained from the same amount of *E.coli* cells, transformed with empty pET28a vector, were incubated for 30 min with 10 nM ^3^H-cAMP and assayed for ^3^H-cAMP hydrolysis. D. *Mg^2 +^ dependence.* Purified *Acas* RegA was incubated with 10 nM ^3^H-cAMP and increasing concentrations of MgCl_2_ and assayed for ^3^H-cAMP hydrolysis. Data are expressed as percentage of ^3^H-cAMP hydrolysis occurring at 0.3 mM MgCl_2._ E. *Substrate* s*pecificity.* Purified *Acas* RegA was incubated with 10 nM ^3^H-cAMP and increasing concentrations of cAMP and cGMP, and assayed for ^3^H-cAMP hydrolysis. Data are expressed as percentage of hydrolysis at 10 nM ^3^H-cAMP only. Means and SD of two experiments performed in triplicate are presented. F. The data for competition by cAMP (panel E) were converted into moles of 5′AMP produced per μg protein per min (V) at each concentration (S) and plotted as S/V against S in a Hanes plot. Intersections of the plot with the abscissa and ordinate, represent − K_M_ and K_M_/V_max_ values, respectively, yielding a K_M_ of 19 μM and a V_max_ of 55 nmol/min μg protein.

The *Acas RegA* cDNA was fused to a hexahistidine tag in vector pET28a and transformed into *E.coli.* The expressed RegA protein was purified by Ni^+^ chromatography and yielded the expected 70 kD band (Fig. 4A), which, after blotting to nitrocellulose, reacted strongly to an anti his-tag antibody (Fig. 4B). When compared to empty vector controls, the eluates from RegA transformed *E.coli* showed 15-fold higher ^3^H-cAMP hydrolytic activity (Fig. 4C), indicating that *Acas RegA* encodes a cyclic nucleotide phosphodiesterase.

HDc type PDEs require Mg^2 +^ as a co-factor and can also hydrolyse cGMP. We therefore first tested Mg^2 +^ dependence and substrate specificity of *Acas* RegA. The purified enzyme required 0.3 mM Mg^2 +^ for optimal PDE activity (Fig. 4D) and cAMP, but not cGMP, competed with ^3^H-cAMP for hydrolysis (Fig. 4E), indicating that *Acas* RegA specifically hydrolyses cAMP. Conversion of the competition data into a Hanes plot [47] yielded an apparent K_M_ of 19 μM and V_max_ of 55 nmol/min μg protein (Fig. 4F). From an estimated 60% purification of the 70 kD RegA protein, the V_max_ translates into a k_cat_ of 60 s^− 1^.

### 3.4 *Acas* RegA inhibitor selection and the effects of inhibitors on encystation

**Fig. 5:**
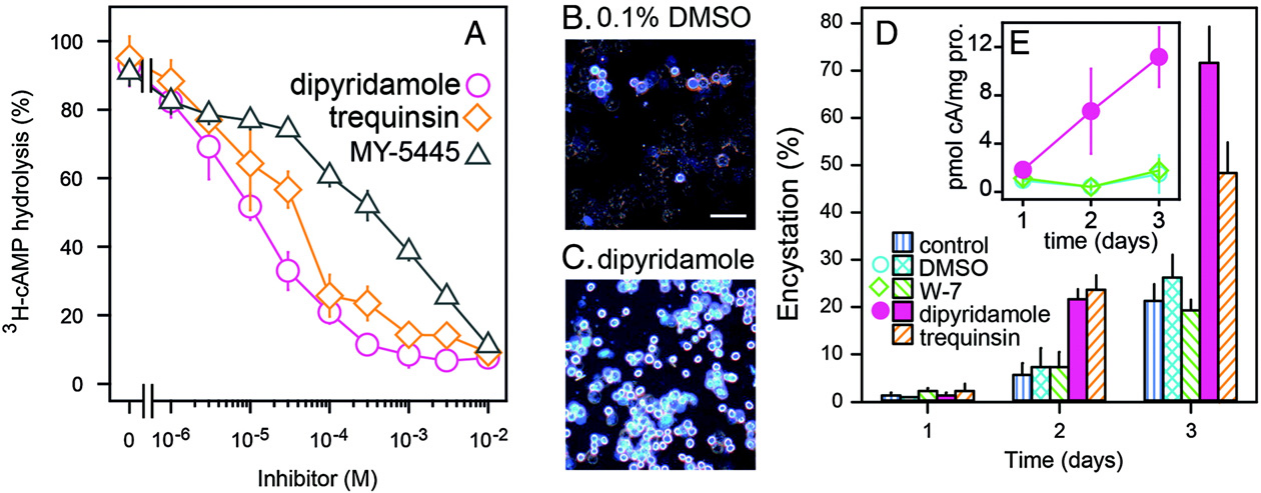
**Selection of *Acas* RegA inhibitors and their effects on encystation**. A. *Inhibition.* Three compounds that inhibited *Acas* RegA activity at concentrations < 100 μM were selected from a panel of 32 PDE inhibitors (Table S2) and tested at a concentration range of 10^− 6^ to 10^− 2^ M for inhibition of ^3^H-cAMP hydrolysis. Means and SD of 2 experiments performed in triplicate are presented. *B–D. Encystation. Acas* amoebas were resuspended in starvation buffer (SB) and incubated with 100 μM of the effective *Acas* RegA inhibitors dipyridamole and trequinsin, the inactive compound W-7, 0.1% DMSO (the W-7 and dipyridamole solvent) and without additives (control). At successive days, cells were harvested, stained with calcofluor, and photographed under UV and weak phase-contrast illumination to identify cysts and amoebas respectively. B/C. Images of cells incubated for 3 days with 0.1% DMSO (B) or 100 μM dipyridamole (C) and stained with calcofluor, Bar: 10 μm. D. Amoebas and cysts were counted in total sample sizes of 600 cells, and the percentage of encysted cells was determined. Means and SD of 2 experiments are presented. E. *cAMP levels. Acas* cells incubated with dipyridamole, W7 and 0.1% DMSO, as above, were lysed in perchloric acid at the indicated time points and assayed for total cell-associated cAMP levels. Data are standardized on the protein content of the samples and represent means and SD of 2 experiments performed in triplicate.

A range of enzyme specific inhibitors have been developed for mammalian cyclic nucleotide phosphodiesterases and we tested a panel of 32 of these compounds for inhibition of *Acas* RegA at concentrations of 30, 100 and 300 μM (Table S2). Three compounds, dipyridamole, MY-5445 and trequinsin, inhibited *Acas* RegA at concentrations below 100 μM. The concentration dependence of these compounds for inhibition of *Acas* RegA was examined in greater detail (Fig. 5A) and yielded IC_50_ values of 400 μM for MY-5445, 30 μM for trequinsin and 12 μM for dipyridamole.

The most effective inhibitors, dipyridamole and trequinsin were subsequently tested for effects on *Acas* encystation, using W-7, a PDE inhibitor that did not inhibit RegA (Table S2) and the solvent 0.1% DMSO as controls. Upon starvation, *Acas* amoebas normally start to encyst after two days. However, encystation was strongly accelerated in the presence of 100 μM dipyridamole or trequinsin, with the most effective compound, dipyridamole, inducing over 70% encystation within three days, when the three controls only showed 20–25% encystation (Fig. 5B-D). Trequinsin, but not W-7, increased cAMP levels from 1 to 11 pmol/mg protein during the experiment (Fig. 5E), indicating that trequinsin stimulates encystation by inhibiting cAMP hydrolysis by RegA.

## 4 Discussion

### 4.1 RegA genes are deeply conserved

Activation of PKA by cAMP controls almost all aspects of the *Ddis* life cycle, starting with the transition from growth to aggregation [48] to the differentiation of prespore cells [18], the maturation of spores and stalk cells [49] and the control of spore dormancy [23]. While PKA activity requires cAMP synthesis by several adenylate cyclases, it is actually RegA that controls PKA activity by integrating the external stimuli that regulate its cAMP hydrolytic activity. These stimuli, such as solute stress, NH_3_, spore-inducing peptides and cytokinins are detected by sensor histidine kinases/phosphatases [27], [28], [29], [50], which target the phosphoryl accepting aspartate residue in the response regulator of RegA [51].

Within Dictyostelia, the *Ppal* and *Ddis* lineages separated from each other at least 0.5 billion years ago [40]. Dictyostelids and Acanthamoebids are members of Conosa and Lobosa, respectively, the two major subdivisions of the protist kingdom Amoebozoa [52], while *Naegleria gruberi* is a member of another protist kingdom, Excavata [53]. While these lineages diverged at the earliest origins of eukaryotes [53], the dictyostelid, *Acas* and *Naegleria* genomes all contain a conserved *RegA* gene and, similar to Dictyostelia [39], [40], [41], the *Acas* and *Naegleria* genomes also contain a large number of sensor histidine kinases [43], [44], the upstream regulators of the RegA. PKA catalytic and regulatory subunits, the downstream target of RegA, are also present in all genomes, indicating ancient origins for histidine kinase regulated cAMP signalling. Neither *Acas* nor *N. gruberi* have multicellular development and also only 6 of the 15 Dictyostelid sensor histidine kinases have been assigned roles in multicellularity. It is therefore likely that ancestrally these sensors detected environmental signals and that their role in developmental signalling evolved only recently in Dictyostelia.

### 4.2 RegA controls multicellular development and encystation in the Dictyostelid *P.pallidum*

*Ddis* and all other dictyostelids that use cAMP as chemoattractant for aggregation are members of taxon group 4. Members of the other three groups use other attractants and unlike group 4 species, many of these species have retained the ability to encyst [54]. We show that in *Ppal*, the only genetically tractable member of this set, loss of RegA results in accelerated multicellular development (Fig. 2) as is also the case in *Ddis* [24]. However, the *Ppal rega1-*cells also aggregated precociously while feeding on bacterial lawns, which was not reported for *Ddis*. Most strikingly, when grown in liquid media, where cells cannot aggregate, the *Ppal rega1-*cells fully encysted in the absence of nutrient stress, essentially inhibiting proliferation under this culture condition (Fig. 3). Loss of RegA1 also strongly inhibited the germination of spore and cysts, indicating that germination requires reduced cAMP levels and loss of PKA activity.

We already reported earlier that PKA activation by ACG is required for *Ppal* encystation [55]. The present work shows that the role of RegA is to prevent encystation in the absence of stress, and to promote cyst (and spore) germination when conditions for proliferation are favourable.

### 4.3 RegA controls encystation in *Acanthamoeba castellani*

Cloning and heterologous expression of the *Acas RegA* gene showed that it also encodes a cAMP phosphodiesterase (Fig. 4). From a panel of 32 mammalian PDE inhibitors, two compounds, trequinsin and dipyridamole, appeared to inhibit *Acas* RegA effectively (Fig. 5). Similar to genetic abrogation of *RegA* in *Ppal*, pharmacological inhibition of RegA in *Acas* strongly stimulated encystation, with RegA inhibition causing a 10-fold increase in cellular cAMP levels. These data strongly suggest that *Acas* encystation is also induced by elevated cAMP and that RegA plays a crucial role in regulating cAMP levels. There is sporadic evidence that the PKA agonist dibutyryl cAMP triggers encystation in the Amoebozoa *Hartmannella culbertsoni* [56] and *Entamoeba invadens* [57], but no proteins for detection, synthesis or degradation of cAMP were previously reported.

### 4.4 Conclusions

• The resilient properties of cysts from pathogenic amoebas such as *Acanthamoeba castellani* prevent successful treatment of vision destroying *Acanthamoeba* keratitis.
• By combining gene knockout studies with pharmacological intervention, we show that intracellular cAMP mediates stress-induced encystation in Amoebozoa and that RegA critically regulate cAMP levels in this process.
• These findings lead the way to screens for compounds that interfere with cAMP synthesis or signal transduction in Amoebozoa and consequently for development of therapeutics that render amoebozoan pathogens susceptible to immune attack and conventional antibiotics.
