# Supplementary material for: The cyclic AMP phosphodiesterase RegA critically regulates encystation in social and pathogenic amoebas
Source: Cell Signal. 2014 Feb;26(2):453–9. doi: 10.1016/j.cellsig.2013.10.008 (PMC3906536; doi:10.1016/j.cellsig.2013.10.008)
Supplement: Supplementary file 1 — Supplementary figures S1 and S2; supplementary tables S1–S3. [file mmc1.doc]

**SUPPLEMENTARY MATERIAL**

Supplementary figures S1,S2; supplementary tables S1-S3

**Figure S1**

**A. PDE domain**

**...AA...M...................................MM.W......................**

**PDE4b2**  **DVAYHNSLHAADVAQSTHVLLSTPALDAVFTDLEILAAIFAAAIHDVDHPGVSNQFLINTNSELALMYND**

**DdisRegA**  **NNRYHNFTHAFDVTQTVYTFLTSFNAAQYLTHLDIFALLISCMCHDLNHPGFNNTFQVNAQTELSLEYND**

**DpurRegA**  **NNRYHNFMHAFDVTQIVYTFLTSFNAVQYLTHLDVLALLISCMCHDLNHPGFNNTFQVNAQTELSLEYND**

**DlacRegA**  **SNRYHNFIHAFDVTQTCYSFLTSFNAAQYLTHLDILSLLISCMCHDLNHPGFNNAFQVNSQSELSMTYND**

**PpalRegA1** **SNRYHNFLHAFDVTQTCYTFLTTFKAAQYLTHLDILSLLIAAMCHDLNHPGFNNTFQVNAQTDLTLQYND**

**PpalRegA2** **DHPYHNFYHAFDVTQTCFSYLTTFGLSHYLTHMDIITILLCALCHDLNHPGFNNAFMVNTFDPIAMQYND**

**DfasRegA1** **NNRYHNFMHAFDVTQTCYTFLTSFKAAEYLTHLDILSLLIASMCHDLNHPGFNNTFQVNAQTELSLQYND**

**DfasRegA2** **GNRYHNFIHAFDVTQTCYSLLTKFKAAEYLTHLDILSLLISSLCHDLDHPGVNNTFQINAQTELSLKYND**

**AcasRegA**  **KNPYHNFRHAFDVTHCCYLVLTSGGAMELVTHLEIFALLISAICHDFEHPGLNNTFLANTSNSLALRYND**

**NgruRegA**  **HNPYHNFTHAVDVTQFAYHLLLVDRISQMFSPMEKFALMFSAIMHDVGHPGVNNNYLINIKDELALIYND**

**...W...W.....................................W.A......................**

**PDE4b2**  **ESVLENHHLAVGFKLLQEEHCDIFMNLTKKQRQTLRKMVIDMVLATDMSKHMSLLADLKTMVETKKVTSS**

**DdisRegA**  **ISVLENHHAMLTFKILRNSECNILEGLNEDQYKELRRSVVQLILATDMQNHFEHTNKFQHHLNNLPFDR-**

**DpurRegA**  **ISVLENHHAMLTFKILRNSECNILEGLNEDQYKELRRSVVQLILATDMQSHFEYINKFQHHLNNLPFDR-**

**DlacRegA**  **VSVLENHHAMLTFKILKNSECNILEGLNEDQYKELRRSVVQLILATDMANHFEHISKFQHHLNSSEFDR-**

**PpalRegA1** **NSVLENHHATLTFKILKNSDCNILEGLNEDQYKELRRSVIQLILATDMAFHFEYINKFQHHLNNQPFDR-**

**PpalRegA2** **RSVLENHHLSLMWKLMDQ--FNILSALSRDELKEFRQIAITCIISTDMSLHFPLVTRLKQRLTELKLNNL**

**DfasRegA1** **NSVLENHHATLTFKILKNNDCNILEGLNEDQYKELRRSVIQLILATDMSFHFEYINKFQHHLNNQPFDR-**

**DfasRegA2** **NSVLENHHARLAFKILRDPDCNILEGLSESQYREIRRSIIQLILSTDMSFHYEYINKFQNHLSQQPFDR-**

**AcasRegA**  **RSILENHHCARAFLLMRKPETEILTGLTDLEYRELRKIVVNCILSTDMLKHVEIVTKFTTMVDQFTRE--**

**NgruRegA**  **VSVLENHHASQAFYLLLK--HNICSNLSKDEFKEFRRLVISTILCTDMSHHFEILTKFQTRLQTGTLSK-**

**......................MA.AA......A......A..AA.........................**

**PDE4b2**  G**VLLLDNYTDRIQVLRNMVHCADLSNPTKSLELYRQWTDRIMEEFFQQGDKERERGMEISPMCDKHTASV**

**DdisRegA**  **-----NKKEDRQMILNFLIKCGDISNIARPWHLNFEWSLRVSDEFFQQSHYETICGYPVTPFMDKTKTTR**

**DpurRegA**  **-----NKKEDRQMILNFLIKCGDISNIARPWHLNFEWSLRVSDEFFQQSHYETICGYPVTPFMDKTKTTR**

**DlacRegA**  **-----NKKEDRQQILNFLIKCGDISNVARPWELNFEWSIRVSDEFFQQSQYEKVCGFPVAPFMNKTQTTR**

**PpalRegA1** **-----NKKEDRQMILNFLIKCGDISNVARPWHLNLEWSNRVSDEFFQQSQFEKVCGYPVTPFMDKTKTNR**

**PpalRegA2** **-KSLDNSLQDRIFLMEIMLHAADISNVSKPWDVSKIWCHRINVESFVQGRMEHLKGIPVTDWMDETKTSV**

**DfasRegA1** **-----NKKEDRQMILNFLIKCGDISNVARPWHLNYEWSIRVSDEFFQQSNFEKVCGYPVTPFMDKTKTTR**

**DfasRegA2** **-----NKSEDRQLLLIYLIKCGDISNVAKPWPLNHQWSNRIADEFFEQASFERIRNLPVAPFMDKNQTTK**

**AcasRegA**  **------NREHRALLLEIILKCADISNPTRPPRIAAYWSQMVQEEFFAQGDKEKEEGLPVSPFMDRDNSLP**

**NgruRegA**  **-----ESKEDRLQLMGVILKCSDVSNALRPFDVSEKWSNVLLEEFFLQGDSERDRGLPISPLMDRRSVDK**

**...A..A........**

**PDE4b2**  **EKSQVGFIDYIVHPL**

**DdisRegA**  **ARIAADFIDFVASPL**

**DpurRegA**  **ARIAADFIDFVASPL**

**DlacRegA**  **ARIAADFIDFVALQL**

**PpalRegA1** **PRIAADFIDYVALPL**

**PpalRegA2** **EKNSLNFMVYLGSAF**

**DfasRegA1** **PRIAADFIDLVALPL**

**DfasRegA2** **PRIAADFIDYVAGPL**

**AcasRegA**  **GKMVVGFVDFFVAPL**

**NgruRegA**  **PKSQLNFIDYIAAPL**

**B. Response regulator domain**

**..........AA................................................P.........**

**EcolCheY**  **ADKELKFLVVDDFSTMRRIVRNLLKELGFNNVEEAEDGVDALNKLQ-----AGGYGFVISDWNM--PNMD**

**ScerSLN1**  **--TSVKILVVEDNHVNQEVIKRMLNLEGIENIELACDGQEAFDKVKELTSKGENYNMIFMDVQM--PKVD**

**DdisRegA**  **SPSKVRILVADDDDVQRKILNNLLKKFHYNV-TLVPNGEIAWEYIN---KGQQKYDLVLTDVMM--PHIT**

**DpurRegA**  **SPSKVRILVVDDDDVQRKVLENLLKKFHYNV-TLVSNGEIAWDNIS---SGQQKYDLVLTDVMM--PHIT**

**DlacRegA**  **SPSRVHILVADDDVIQRKILENVLKKFHYNV----HNGEEAWECLE---KGQVKYDLVLTDVMM--PEIN**

**PpalRegA1** **SPSKVHILVVDDDIVQRSLLNNMLKKFNFNV-TLVTNGEEAWDTLL---NGKVMYDLVLTDVMM--PMVT**

**PpalRegA2** **INKETKFLIIDFDNKSRELLEIWLRTEGFNV-FSSKNVVDSLSLLE-----NNSYDLVIYDTDSKIENIE**

**DfasRegA**  **SPSKVHILVVDDDQVQRKILESALKKFKYNV-TVVTNGEEAWNILI---NGNTKYDLVLTDVMM--PNIS**

**DfasRegA2** **CPSSIHILVVDDDITQRKVLQNLLSKVFYNV-TLVSSAEEAWNMLI---YGTTKFDLVLTDVMM--PQVT**

**AcasRegA**  **HPKEIKILVVDDEPTARIVVRKLLEKTGYTEVEVVESGRKAIELIE-----NRSFNLVLCDLHM--PDID**

**NgruRegA**  **DEGAKKVLVVDDDRVARQVLTKLLEQLGFQV-KAVEGGKQALEVLR---EEGNNYHLLLVDVLM--PDMD**

**......................S...................S..A........................**

**EcolCheY**  **GLELLKTIRADGAMSALPVLMVTAEA-KKENIIAAAQAGASGYVVKPFTAATLEEKLNKIFEKLGM----**

**ScerSLN1**  **GLLSTKMIRRDLGY-TSPIVALTAFA-DDSNIKECLESGMNGFLSKPIKRPKLKTILTEFCAAY-QGKKN**

**DdisRegA**  **GFDLLQRINDHPVHRHIPVILMSGTAVDYKYANDTIKIGGQDFLTKPIAKELLKKKIDTVLQSIWQRRKE**

**DpurRegA**  **GFDLLQRINDHPIYKNIPVILMSGTAVDYKYANDTIKIGGQDFLTKPIAKELLKKKIDTVLQSIWQRKKE**

**DlacRegA**  **GFDLCTKINLHPVFNKIPVILMSGTAVDYKCANDTIKIGGQDFLTKPIAKELLKKKIDTLLGSIWQKKKE**

**PpalRegA1** **GFDLLQRINEHIEIKHIPVILMSGTAVDYKYANDTIKIGGQDFLTKPIAKELLKKKIDTVLTSIWQKKKE**

**PpalRegA2** **TLTNSQIFYIVNKMEFSQF--------------QQKSNNKLEFQSKPVSKGNLLSHITYVLENRLKSYKL**

**DfasRegA**  **GFDLLQRINDHAEIKNVPVILMSGTAIDYKYANDTIKIGGQDFLTKPIAKELLKKKIDTVLNSILREKNE**

**DfasRegA2** **GFDLLQRINEHPEIKNIPVILMSGTALDCKYANDTIKIGGQDFVTKPIAKELLKKKIDILLRSKHQKKQE**

**AcasRegA**  **GIGVVKAVRKKAHMDDSPIVMMSATE-DLNIVYKCLSEGADDYLLKPIQANAVKNLW----QNVWRKRKE**

**NgruRegA**  **GLQLLKIFRQ-AYSEDMPIIMVSSSE-DPDTINQCFQSGAEDFLQKPVQLEILKRRVNMCLEDRLRRRKE**

**Figure S1. Alignments of the PDE and response regulator domains of RegA with structurally resolved homologs**

*A. PDE domain.* HDc-type PDE domains of *Ddis* RegA homologs were aligned with the structurally resolved cAMP phosphodiesterase, Pde4b2. Residues that were identical or similar in 70% of sequences are shaded in black or grey, respectively. Red highlight: residues that coordinate the Me^2+^ ions (“M” above sequences), Blue highlight: residues that form a water-mediated interaction with the Me^2+^ ions (W). Green highlight: residues that interact with the substrate, or are otherwise involved in catalysis . Genbank IDs: Ddis RegA: XP_638612; Dpur RegA: XP_003285505; Dlac RegA: KC012946; Ppal RegA1: EFA83871; Ppal RegA2: EFA76700; Dfas RegA1: EGG23932; Dfas RegA2: EGG17246; Acas RegA: KC012947; Protein Data Bank code: PDE4b2: 1ROR.

*B. Response regulator domain.* The response regulator (RR) domains of *Ddis* RegA homologs were aligned with the structurally resolved RR domains of *E.coli* CheY and *Saccharomyces cerevisae* SLN1. Black and grey highlights mark amino acid residues with identity or similarity across ≥ 70% of sequences, respectively. Red: phosphoryl accepting aspartate (P); Blue: residues in the Mg^2+^-binding active site (A); Maroon, switch-pair residues that re-orient upon aspartate phosphorylation (S) . Protein Data Bank codes: EcolCheY: 1F4V; ScerSLN1: 2R25.

**Figure S2**


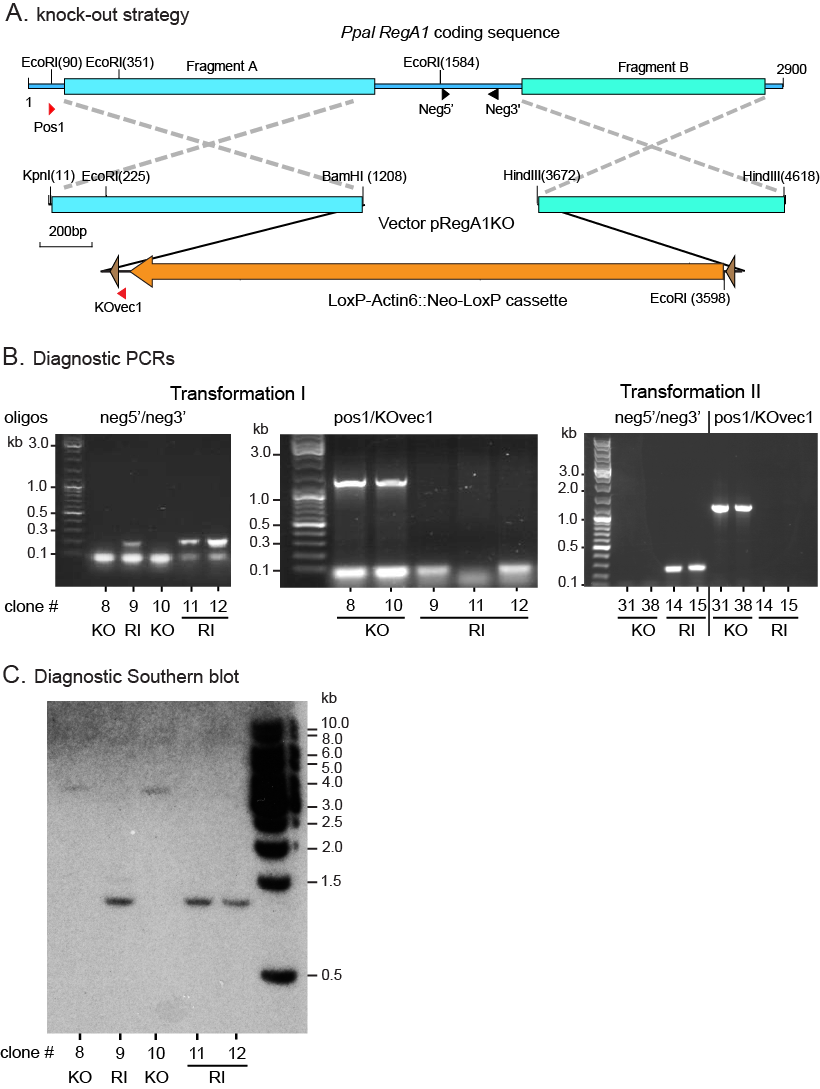


**Figure S2. *Ppal* RegA1 gene disruption strategy and validation**

*A. Knock-out strategy.* Two *Ppal RegA1* fragments were amplified from genomic DNA and cloned into vector pLoxNeoI to flank the floxed actin6-neo cassette. After homologous recombination, 562 bp of the RegA coding sequence, including more than 1/3^rd^ of the PDE catalytic domain will be replaced by the floxed actin6-Neo selection cassette. The positions of primer pairs Neg5’/Neg3’ and Pos1/KOvec1 that are used to diagnose a negative and positive events of homologous recombination, respectively, are indicated.

*B. PCR diagnosis.* Genomic DNAs of G418 resistant clones from two independent transformations (I and II) of *Ppal* cells with the pRegA1KO vector were amplified with primer pair Neg5’/Neg3’ that should yield a 0.22 kb product in wild-type cells and random integrants, and primer pair Pos1/KOvec1 that should yield a 1.25 kb product, after homologous recombination. Clones 8, 10, 31 and 38 were identified as RegA1 knock-outs (KOs) and clones 9, 11, 12, 14 and 15 as random integrants (RI).

C. *Southern blot.* Genomic DNAs of the clones 8-12 were digested with EcoRI, size fractionated, transferred to nylon membrane and probed with the 5’-*Kpn*I/*Bam*HI-fragment of the pRegA1KO vector. Wild-type and RI DNAs should yield a band at 1.2 kb, while KOs should yield a band at 3.4 kb.

**Table S1. Oligonucleotide primers used in this work**

| Name | Restric-tion sites | DNA sequence |
| --- | --- | --- |
| PpRegAI5’ | KpnI | GGGGTACCCGCGATAAGCAGTGTCAGTGTCACCTTG |
| PpRegAI3’ | BamHI | CGGGATCCGGTTTGTTGCATCAGTTCTTCGATGG |
| PpRegAII5’ | HindIII | CCCAAGCTTGCAATACCATTGCCACAATCGATTCTTCTAG |
| PpRegAII3’ | HindIII | CCCAAGCTTGGGTGAATCGGTGGGTGAGTTGGGCG |
| RegAneg5’ |  | TCGTCGGTGACAGGCGCCCGAAGAAAC |
| RegAneg3’ |  | GCCATAATAAATCTCTGTAGTTTTTCGATGGGAATCT |
| RegApos1 |  | CCGGGCCCCCGATCAGAAGAATTCCGGTG |
| KOvec1 |  | GGGCGATCGGTGGCGGGGATCATCATAACTT |
| AcRegAF | NheI | ATGCTAGCATGCCGGATATCGACGGCAT |
| AcRegAR | EcoRI | AGGAATTCCTACTCAGCCTTGGCGCTGC |

**Table S2. Effects of phosphodiesterase inhibitors on *A.castellani* RegA activity**

| **PDE inhibitor** | | **PDE inhibitor** | |
| --- | --- | --- | --- |
| **Trade name** | **inhibits?** | **Trade name** | **inhibits?** |
| Dipyridamole | strongly | CGH 2466 | slightly |
| Vinpocetine | slightly | Anagrelide | moderately |
| Rolipram | no | Irsogladine | no |
| Cilostamide | slightly | CP 80633 | no |
| Zaprinast | slightly | Caffeine | no |
| Zardaverine | slightly | Theophylline | slightly |
| Siguazodan | no | IBMX | moderately |
| EHNA | moderately | Mesopram | no |
| (R)-(-)-Rolipram | no | Pentoxifylline | no |
| (S)-(+)-Rolipram | no | RS 25344 | slightly |
| Milrinone | no | Sildenafil | slightly |
| T 0156 | no | W-7 | no |
| Cilostazol | moderately | W-9 | slightly |
| Ibudilast | moderately | A-7 | no |
| ICI 63,197 | no | Ro 20-1724 | moderately |
| YM 976 | moderately | MY-5445 | strongly |
| BRL 50481 | slightly | Etazolate | slightly |
| Trequinsin | strongly | MMPX | no |

PDE inhibitors at 0, 30, 100 and 300 µM were incubated for 30 min with recombinant purified *Acas* RegA and ^3^H-cAMP and scored for inhibition of ^3^H-AMP production. Inhibitory effects observed at 300 µM are qualitatively listed in Table S2. The three most active compounds dipyridamole, trequinsin and MY-5445 were then tested over a broader concentration range (Fig. 5A). All inhibitors were purchased from Tocris Bioscience (Bristol, UK).

**Table S3. Culture media and buffers**

| Name | Content per litre water |
| --- | --- |
| 1/5^th^ SM agar | 2 g glucose, 2 g Bacto peptone, 2.2 g KH_2_PO_4_, 1.25 g Na_2_HPO_4_.2H_2_O and 15 g agar, pH 6.5. |
| HL5 | 14.3 g Difco proteose peptone, 7.15 g yeast extract, 16 g glucose, 0.626 g Na_2_HPO_4_.2H_2_O, 0.485 g KH_2_PO_4_, pH 6.5. |
| AC | 15 g glucose, 7.5 g yeast extract, 7.5 g Difco proteose peptone |
| SB | 100 mM KCl, 8 mM MgSO_4_, 0.4 mM CaCl_2_, 1 mM NaHCO_3_, 20 mM Tris-HCl pH 8.8 |
| NN agar | 15 g agar, 1.2 g KH_2_PO_4_, 0.48 g Na_2_HPO_4_.2H_2_O, pH 6.5 |

**REFERENCES**

[1] Xu RX, Rocque WJ, Lambert MH, Vanderwall DE, Luther MA, Nolte RT, Crystal structures of the catalytic domain of phosphodiesterase 4B complexed with AMP, 8-Br-AMP, and rolipram. J. Mol. Biol. 2004;337:355-365.

[2] Gao R, Mack TR, Stock AM, Bacterial response regulators: versatile regulatory strategies from common domains. Trends in biochemical sciences. 2007;32:225-234.
